# Sub-5 nm Gate-Length Monolayer Selenene Transistors

**DOI:** 10.3390/molecules28145390

**Published:** 2023-07-13

**Authors:** Qiang Li, Xingyi Tan, Yongming Yang, Xiaoyong Xiong, Teng Zhang, Zhulin Weng

**Affiliations:** 1 College of Intelligent Systems Science and Engineering, Hubei Minzu University, Enshi 445000, China; 2009006@hbmzu.edu.cn (Y.Y.); 2002009@hbmzu.edu.cn (X.X.); hbmyzt@hbmzu.edu.cn (T.Z.); 1994006@hbmzu.edu.cn (Z.W.); 2 Department of Physics, Chongqing Three Gorges University, Chongqing 404100, China; tanxy@sanxiau.edu.cn

**Keywords:** monolayer selenene, sub-5 nm gate length, density functional theory, quantum transport simulation

## Abstract

Two-dimensional (2D) semiconductors are being considered as alternative channel materials as silicon-based field-effect transistors (FETs) have reached their scaling limits. Recently, air-stable 2D selenium nanosheet FETs with a gate length of 5 µm were experimentally produced. In this study, we used an ab initio quantum transport approach to simulate sub-5 nm gate-length double-gate monolayer (ML) selenene FETs. When considering negative-capacitance technology and underlap, we found that 3 nm gate-length p-type ML selenene FETs can meet the 2013 ITRS standards for high-performance applications along the armchair and zigzag directions in the 2028 horizon. Therefore, ML selenene has the potential to be a channel material that can scale Moore’s law down to a gate length of 3 nm.

## 1. Introduction

The short channel effect of silicon field-effect transistors (FETs) exceeds the physical limits of FETs, making them difficult to apply [1,2,3]. Therefore, the search for a new material to replace silicon is crucial. When successfully foliated, two-dimensional (2D) semiconductors have begun competing with graphene as a future channel material [4,5]. The newly created 2D Xenes (silicene, phosphorene, tellurene, and so on) have the potential to surpass the limitations in practical applications because of their uniform thickness, higher electrostatic gate control, simple elemental composition, and other properties [6,7]. The on/off-state current ratio of sub-10 nm gate length (L_g_) monolayer and bilayer tellurene FETs with p-type characteristics is 10^8^ and 10^6^, respectively [8,9]. The p-type monolayer (ML) tellurene transistor meets the criterion for the International Technology Roadmap for Semiconductors (ITRS), with a gate length of 4 nm [9]. Thus, investigating 2D monoelemental semiconductors, which have good carrier mobility and air stability for use in logic circuits, is required [6,7,9,10,11,12,13,14,15,16].

Selenium, which is known for its essential role in biological systems, has received attention in the realm of physical sciences [17,18,19,20,21,22]. The thermal properties of 2D α-selenene were investigated by Liu et al., revealing its remarkable thermophysical characteristics. At a temperature of 300 K, 2D α-selenene exhibited an excellent thermal conductivity of 3.04 W/(m·K). The study by Liu et al. further emphasized the crucial role played by relaxation time in controlling the thermal transport behavior of 2D α-selenene [23]. Lin et al. conducted a detailed investigation into the thermoelectric properties of 2D ML square selenene, using a comprehensive approach to reveal a significant anisotropy in the thermal performance of the material. These findings provided valuable insights into the complex relationship between directionality and the thermal transport behavior of ML square selinene [22]. 

The photonic properties of 2D selenium nanosheets have also been investigated, revealing remarkable findings regarding the photoresponsivity and temporal characteristics of selenium nanosheet phototransistors. The photoresponsivity measurement has achieved an impressive value of 263 A/W, accompanied by the rapid rise and fall times of 0.1 s and 0.12 s, respectively [17]. These results indicate that ML square selenene is a potential material for thermoelectric and photonic devices in the future. 

Back-gated FETs were fabricated by employing p-type selenium nanosheets with 5 μm L_g_, using the physical vapor-deposition technique. The resulting FETs demonstrated a low on-state current (I_on_) of approximately 20 A/m at V_dd_ = 3 V, which can be attributed to the presence of zigzag edges in the selenium nanosheets. Selenene, a 2D form of selenium, is fabricated in such a way that it is atomically thin [17]. Nevertheless, there is currently a knowledge gap in the existing literature about the fabrication and characterization of sub-5 nm L_g_ selenene FETs (sub-5 FETs).

This study presents a theoretical evaluation of the performances of sub-5 FETs using ab inito quantum transport simulations. The ITRS high-performance (HP) criteria were met by the I_on_ of the 3 nm L_g_ p-type selenene FETs (p-type 3 FETs) with underlap (UL) along the armchair direction (arm-direction) and the I_on_ of the 1 nm L_g_ p-type selenene FETs (p-type 1 FETs) with UL were approximately 17.55% and 1.90%, respectively, for the ITRS HP criteria along the armchair and zigzag directions (the arm-direction and the zig-direction). After considering the negative-capacitance (NC) effect and UL, the p-type 3 FETs met the ITRS HP criteria along both the arm-direction and the zig-direction. These results demonstrate that ML selenene is a potential channel material that is capable of extending Moore’s law by 3 nm.

## 2. Result and Discussion

### 2.1. Device Structure

**Figure 1:**
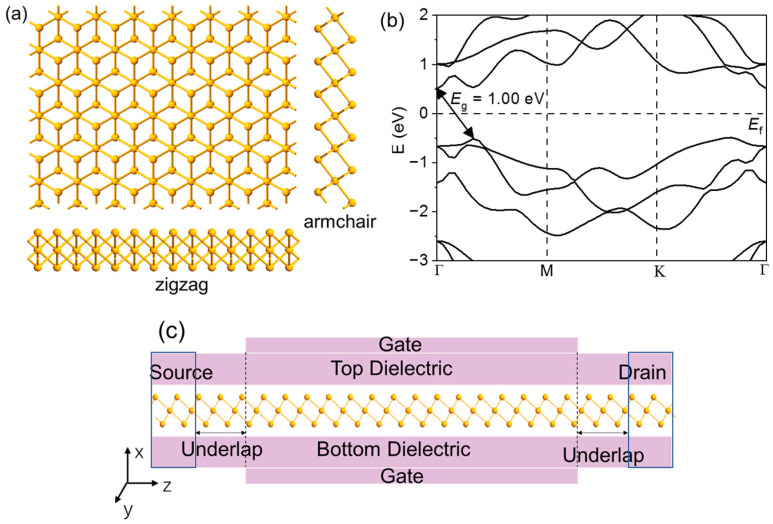
(**a**) Lattice structure of ML selenene (top and side view). (**b**) Band structure of ML selenene. (**c**) Schematic view of the ML selenene FETs.

The optimal lattice parameters of ML selenene are a = 3.76 Å, c = 6.52 Å, based on previous theoretical work [19,20]. Lattice structures of ML selenene from both top and side views are presented in Figure 1a. Figure 1b depicts the band structure of ML selenene using a double-zeta polarized (DZP) basis set in QuantumATK, showing an indirect band gap of 1.0 eV and exceeding the expected theoretical value of 0.73 eV (DFT/PBE) by a small amount, which is close to the theoretical value of 1.13 eV (DFT/HSE06) [24,25]. Figure 1c presents the n- and p-doped ML selenene, serving as channels and electrodes for the sub-5-FETs. A UL area was the uncovered part of the channel by the gate electrode, which was added between the source/drain electrode and gate. UL is an effective method to extend the effective channel length [26,27,28]. We executed the test for doping concentration to provide the best possible device performance (Figure S1). According to the test for doping concentration, we found that the off-state current of the 5 nm gate length n-type selenene FETs can not meet I_off_ for ITRS HP along the armchair direction, except for doping concentration n = 5 × 10^12^ cm^−2^, as shown in Figure S1. Thus, the on-state current of the 5 nm gate length p-type selenene FETs is higher than that of n-type selenene FETs. The slope of transfer characteristics curves of the 5 nm gate length p-type selenene FETs is larger than that of n-type selenene FETs. Therefore, the p-type ML selenene FETs perform better than the n-type ones in the device. As a result, we exclusively investigated p-type ML selenene FETs in the arm- and zig-directions, and we adopted the doping concertation to 5 × 10^13^ cm^−2^ in the following calculations.

### 2.2. On-State Current

**Figure 2:**
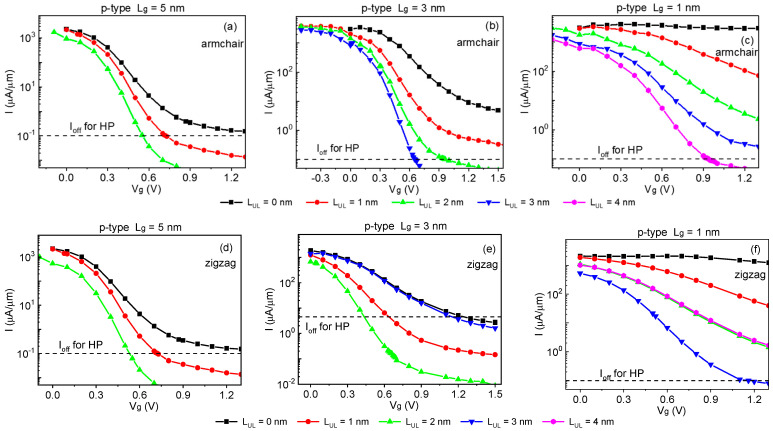
Transfer characteristics of the p-type sub-5 FETs with *L*_UL_ = 0, 1, 2, 3, and 4 nm (**a**–**c**) along the armchair and (**d**–**f**) zigzag direction. The bias is 0.64 V. Black dashed lines represent the ITRS HP requirements for the off-state current.

**Figure 3:**
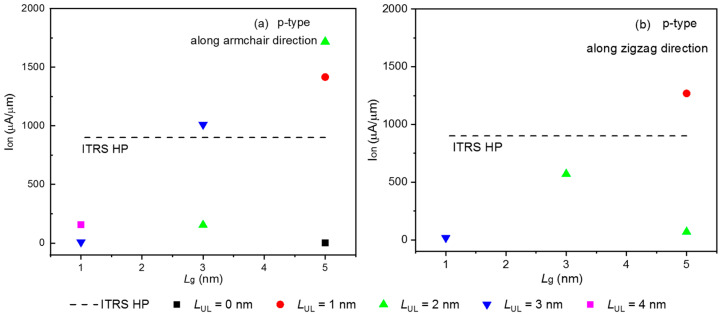
On-state current as a function of the p-type sub-5 FETs with different gate lengths for HP applications along the (**a**) armchair and (**b**) zigzag direction.

A critical figure of merit for the logic transistor is the I_on_. Figure 2 depicts the transfer characteristic curve of the sub-5 FETs along the arm- and zig-directions. According to ITRS 2013, V_dd_ is the power supply voltage, chosen as V_dd_ = 0.64 V, I_off_ = 0.1 μA/μm. We can obtain the off-state voltage (V_g,off_) from transfer characteristics curves of the sub-5 nm gate lengths selenene FETs at the off-state current (I_off_). Thus, the on-state voltage and current (V_g,on_ and I_on_) can be obtained. Figure 2 can determine V_g,on_ = V_g,off_ ±V_dd_ (“+” and “−” sign corresponds to n- and p-types) [29]. The power supply voltage is denoted by V_dd_, according to ITRS 2013, V_dd_ = 0.64 V, I_off_ = 0.1 μA/μm. The transfer current is calculated using the width normalization, and the width is 3.761 Å (6.515 Å) along the arm-(zig-) direction throughout the whole paper. All the p-type sub-5 FETs without UL cannot comply with the ITRS HP requirement along the arm- and zig-directions. After summarizing I_on_ in various L_g_ and UL lengths listed in Table 1 and Table 2, suitable UL lengths could significantly improve I_on_ for both the arm- and zig-directions in the ITRS HP application. The I_on_ of the p-type 3 FETs with *L*_UL_ = 3 nm was 1010.32 μA/μm along the armchair directions, whereas the I_on_ (571.02 μA/μm) of the p-type 3 FETs with *L*_UL_ = 2 nm was 63% of the ITRS for HP goal along the zig-directions, as shown in Figure 3.

**Figure 4:**
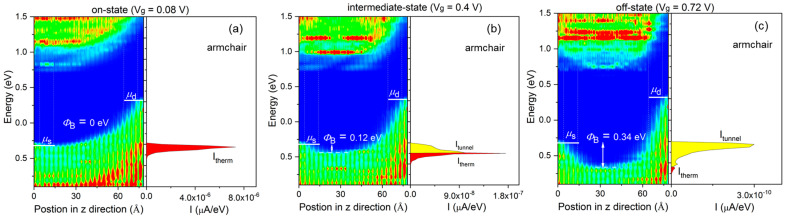

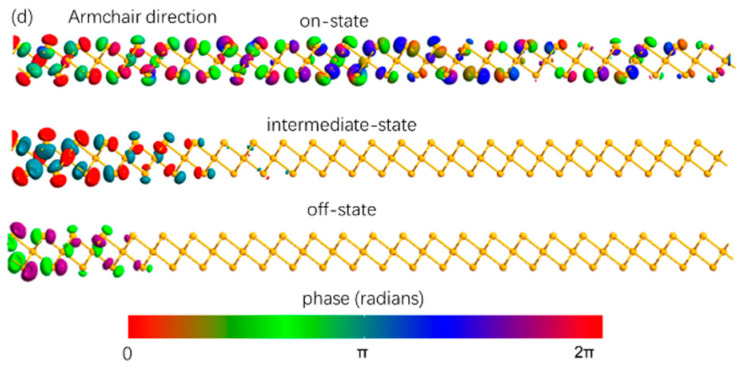
(**a**–**c**) LDOS and spectrum current of the p-type 5 FETs with *L*_UL_ = 1 nm at the on-, intermediate- and off-state for the HP applications along the armchair direction. μ_s(d)_ are the electrochemical potentials of the source (drain), respectively. Φ_B_ is the activation energy height. (**d**) On-, intermediate-, and off-state transmission eigenstates of the p-type 5-FETs with *L*_UL_ = 1 nm at E = −0.38 eV. The isovalue is 0.05 a.u.

Figure 4 presents spectrum characteristics of the 5 nm L_g_ p-type selenene FETs (p-type 5 FETs) with *L*_UL_ = 1 nm in the HP application, allowing us to observe gate modulation along the arm-direction. Examples will be shown to demonstrate the spectrum current and position-resolved local density of states (LDOS) at on-, intermediate-, and off-states. The distance between the source’s Fermi level and the channel’s lowest valence band maximum is known as the hole activation energy (Φ_B_). As V_g_ rises from 0.08 to 0.72 V, the channel’s band edge tilts downwards, increasing from 0 to 0.34 eV. Consequently, the total current (I_total_) in the device comprises tunnel and thermal current (I_tunnel_ and I_therm_). Figure S2 depicts the 5 FETs with *L*_UL_ = 1 nm in the zig-direction. The gate modulation mechanism of the p-type 5 FETs with *L*_UL_ = 1 nm in the zig-direction is similar to that in the arm-direction. The related transmission eigenstates are the transport conditions in the on-, intermediate-, and off-state with E = 0.38 eV. Figure 4 and Figure S2 depict the transmission eigenstates of p-type 5 FETs with *L*_UL_ = 1 nm at E = −0.38 eV in the on-, intermediate-, and off-state, respectively.

### 2.3. Gate Controlling Ability

**Figure 5:**
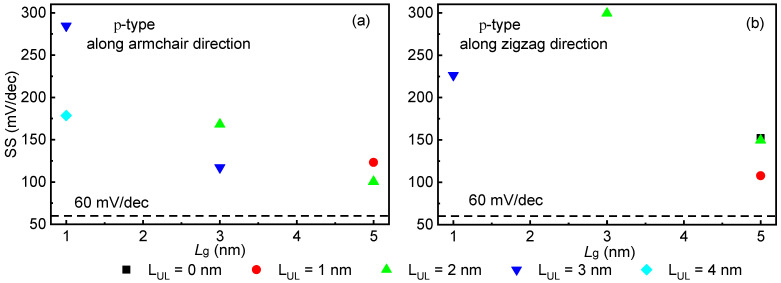
SS versus L_g_ in the p-type sub-5 FETs with different underlap lengths (**a**) along the armchair direction (**b**) along the zigzag direction.

The subthreshold swing (SS = ∂V_g_/∂(logI_ds_)) is crucial in determining the ability of ML selenene FET to gate control [9,14]. We can obtain the SS from transfer characteristics curves of the sub-5 nm gate lengths selenene FETs in the subthreshold zone. A smaller SS offers higher gate control capability in the subthreshold zone. Figure 5 illustrates how the UL structure promotes SS growth as the L_g_ decreases in the p-type 5 FETs. Along the arm-direction, the SS of the p-type 5/3/1 FETs with UL structure is 100.39/117.19/178.68 mV/decade, respectively. The SS of the p-type sub-5 FETs with UL structure along the zig-direction is similar to that of the device in the arm-direction.

### 2.4. τ, PDP and EDP

The switching speed of ML selenene FETs is determined by the effective delay time (τ = C_t_V_dd_/I_on_). C_t_ = C_g_ + C_f_, C_g(f)_ is the gate (fringing) capacitance, and C_g_ = ∂Q_ch_/∂V_g_. According to ITRS 2013, C_g_ is equal to half of C_f_. The QuantumATK 2019 software package was used with the DZP basis set to derive the total Mulliken charge (Q_ch_) in the central region [24]. Furthermore, the C_t_ values of the p-type sub-5 FETs are smaller than the ITRS HP goal along the arm-direction. However, the C_t_ (0.2 fF/μm) of the p-type 3 FETs with *L*_UL_ = 3 nm is smaller than the ITRS HP along the zig-direction, as shown in Figure S3. The C_t_ of p-type 3 FETs along the arm-direction is lower than that of the ones along the zig-direction for the same L_g_. The response speed of the p-type 3 FETs in the arm-direction is faster than the device along the zig-direction, because of combining small C_t_ and large I_on_ to create a high-speed switch.

The switching energy of ML selenene FETs is measured using power dissipation (PDP = V_dd_I_on_τ). Figure S4 shows that the PDP values of the p-type sub-5 FETs decrease with increasing L_g_ along both the arm- and zig-directions. Along the arm-direction, all PDPs of the p-type ML selenene FETs with UL can meet the ITRS HP criteria.

**Figure 6:**
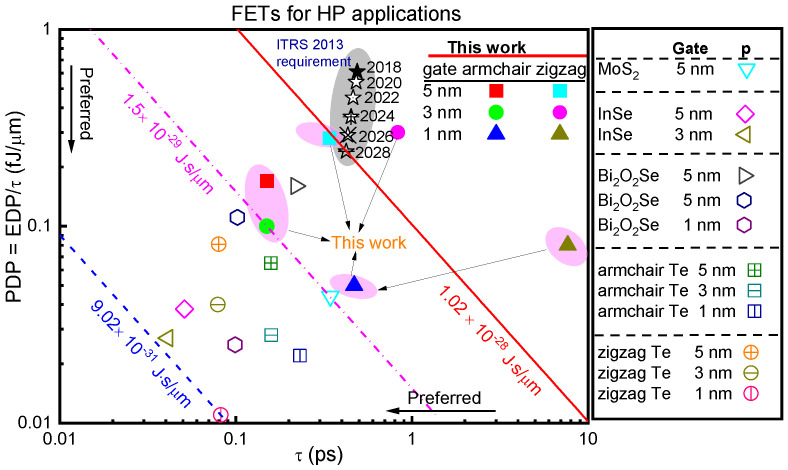
PDP versus τ of ML MoS_2_ [30], InSe [31], Bi_2_O_2_Se [32], and Tellurene FETs (along the arm- and zig-direction) [9] for the HP applications, respectively. Solid lines are the minimum ITRS requirements for the energy–delay product EDP = τ × PDP. Dot and dash dot lines are the minimum EDP value of the p-type sub-5 FETs and ML tellurene FETs reports, respectively.

In Figure 6, we also include the energy–delay product (EDP) of p-type sub-5 FETs. EDP can be defined as EDP = PDP × τ, taking into account both response speed and energy dissipation. The minimum EDP value specified by ITRS requirements, as well as the EDP values for p-type sub-5 FETs and ML Teurene (Te) FETs reports, are plotted in red, pink, and blue lines, respectively. Except for the p-type 3 and 1 FETs with UL along a zig-direction, all of the p-type sub-5 FETs have EDP values that are lower than the EDP of ITRS HP criteria for the 2028 horizon (1.02 × 10^−28^ J·s/μm). The p-type ML selenene FETs have the lowest EDP value at 1.5 × 10^−29^ J·s/μm when L_g_ is set to 3 nm. It can be compared with those of ML MoS_2_ [30], InSe [31], Bi_2_O_2_Se [32], and Tellurene FETs [9]. The EDP of p-type ML selenene FETs is higher than that of ML InSe, Bi_2_O_2_Se, and tellurene FETs. It is roughly 17 times greater than the minimum EDP (9.02 × 10^−31^ J·s/μm) for ML tellurene FETs, but only slightly higher than the minimum EDP (1.34 × 10^−30^ J·s/μm) for ML MoS_2_ FETs.

### 2.5. Discussion

Many 2D transistors are suitable for HP applications, such as ML MoS_2_ [30], ML InSe [31], ML and bilayer (BL) Bi_2_O_2_Se [32,33], ML and BL tellurene [8,9], and ML silicane [15], and so on. Several transport simulations of 2D semiconductors transistors based on the DFT + NEGF method are used to explore the potential channel candidates for post-silicon nanoelectronics. ML MoS_2_ and ML silicane can extend Moore’s law to 5 nm, respectively. ML InSe can extend Moore’s law to 3 nm. ML and BL Bi_2_O_2_Se can extend Moore’s law to 2 and 5 nm, respectively. ML and BL tellurene can extend Moore’s law to 4 and 9 nm, respectively.

**Figure 7:**
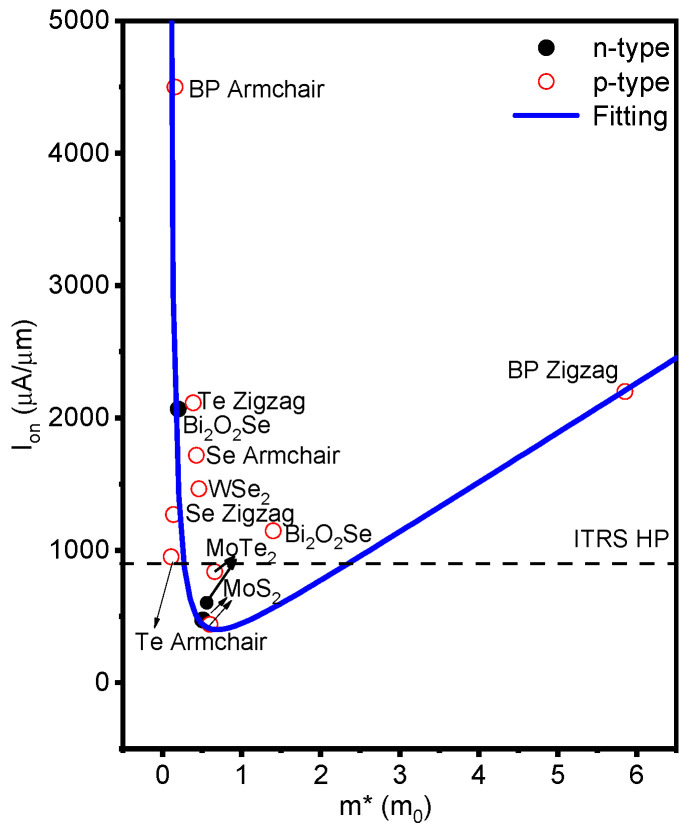
The on-state current versus the effective mass of n- and p-type 5 FETs, and ML 2D channel materials for HP applications. All the data are calculated by ab initio quantum transport simulations. The solid curve line is a visual guide. The dashed line is the ITRS HP requirements of the 2013 edition.

The I_on_ is a critical figure of merit for the logic transistor, to figure out the key factor that affects I_on_, the relationship between the effective mass (m*) and I_on_ of the suggested model at various 5 nm L_g_ 2D semiconductors FETs is displayed in Figure 7 [9,30,31,32]. When m* < 0.68 *m*_0_, I_on_ decreases with increasing m*, and I_on_ is at its lowest when m* ≈ 0.68 *m*_0_. For m* > 0.68 *m*_0_, I_on_ increases with increasing m*. Tiny m* results in a higher I_on_ and greater carrier velocities (v = eEτ/m*), which are determined by the e (charge), E (electric field), and τ, respectively. The current can be written as I = nev, where n is the carrier concentration. The density of states (DOS) of the transport channel is negligibly tiny. DOS is written as $\frac{g_{s}g_{v}}{2\pi \hbar^{2}}\sqrt{m_{x}^{*}m_{y}^{*}}$, where *g_s_*_(*v*)_ is the spin (valley) degeneracies, ℏ is the reduced Plank constant, and $m_{x(y)}^{*}$ is the transverse (transport) effective mass. Larger m* leads to slower velocity, but the DOS of the transport channel is enough; thus, I_on_ is high. According to Figure 7, the competition between m* and DOS results in a dominant factor affecting I_on_. The anisotropy has been found in many 2D materials, such as ML BP [34,35], 2D BiAs [36], ML and BL tellurene [8,9], and ML selinene [19], and so on. The physical properties (elastic modulus, effective mass, deformation potential, and carrier mobility) of ML selenene are different between the arm- and zig-direction. Using the software package QuantumATK 2019, the effective mass (m*) of ML selenene was obtained from the band structure; the hole effective mass of ML selenene is 0.14 *m*_0_ and 0.43 *m*_0_ along the arm- and zig-direction, respectively. It is consistent with previous reports in the literature [19]. The hole effective mass of ML selenene along the arm-direction is lighter than that of the zig-direction, and the I_on_ (1717.80 μA/μm) of p-type 5 FETs along the arm-direction is larger than that (1269.00 μA/μm) of the device along the zig-direction, as shown in Table S1. The I_on_ of ML selenene are different between arm- and zig-direction with 3 nm and 1 nm gate length. Along the arm-direction, the m* (0.14 *m*_0_) of the hole for ML selenene is lighter than that (0.39 *m*_0_) of the ML tellurene, the I_on_ (1717.81 μA/μm) of ML selenene is larger than that (951 μA/μm) of ML tellurene. Along the zig-direction, the effective mass (0.43 *m*_0_) of ML selenene is larger than that (0.11 *m*_0_) of ML tellurene, so the I_on_ (1269 μA/μm) of ML selenene is smaller than that (2114 μA/μm) of ML tellurene.

We added a negative-capacitance (NC) gate stack to the ferroelectric materials to further enhance the performances of the p-type sub-5 FETs in Figure S5a [9,37]. We found that the I_on_ of the device increased, and the SS of p-type sub-5 FETs with the same L_g_ and UL decreased, but the I_on_ of the device increased. It is already known that the NC voltage is V_NC_ = −3.822 × 10^8^ × t_FE_Q + 2.3529 × 10^10^ t_FE_Q^3^ of Hf_0.5_Zr_0.5_O_2_ ferroelectric material [38,39]. The electrical charge is represented by the symbol Q. The thickness of the ferroelectric layer is denoted by t_FE_, and for the ML selenene FETs, we incorporated a ferroelectric layer that was 50 nm thick.

Tables S2 and S3 compare the I_on_ and SS with those without NC for p-type sub-5 FETs with and without NC at *L*_UL_ = 2 nm. Figure S5b compares the arm- and zig-direction transfer properties of p-type sub-5 FETs at L_g_ = 5 nm. Generally, the SS of p-type sub-5 FETs with NC is lower than those of p-type sub-5 FETs without NC. The minimum value of SS (81.26 mV/dec) is obtained by p-type 5 FETs with NC and *L*_UL_ = 2 nm along the arm-direction. The I_on_ of p-type sub-5 nm FETs with NC is greater than those of p-type sub-5 FETs without NC. All I_on_ of p-type 5 FETs and 3 FETs with NC and UL satisfy the 2028 requirements (900 µA/µm) of ITRS HP application along arm- and zig-direction, respectively. The maximum value of I_on_ (3202.95 µA/µm) is obtained by p-type 5 FETs with NC and *L*_UL_ = 2 nm along the arm-direction. In contrast, the maximum value of I_on_ is 3.55 times greater than the ITRS criteria for HP application.

## 3. Model and Approach

Using the software package QuantumATK Version P-2019.03, which combines the density functional theory (DFT) and the nonequilibrium Green’s function (NEGF), the transport characteristics of sub-5 FETs can be determined [24,26,40,41]. The following Landauer–Bűttiker formula is used to determine the drain current at a given bias (gate) voltage *V_b_*_(*G*)_ [41]:

$$I(V_{b},V_{G})=\frac{2e}{h}\int_{-\infty }^{+\infty }\left\{T(E,V_{b},V_{G})[f_{s}(E-\mu_{s})-f_{D}(E-\mu_{D})]\right\}$$

where *e* and *h* are the elementary charges and the Planck constant, respectively, and the electrochemical potential and the Fermi–Dirac distribution function for the source (drain) are denoted by *μ_S_*_(*D*)_ and *f_S_*_(*D*)_, respectively. The transmission coefficient is denoted by *T*(*E*, *V_b_*, *V_G_*). FHI pseudopotential is used with the basis set of polarized double zeta. The exchange–correlation interaction is described using the generalized gradient approximation (GGA) as the Perdew–Burke–Ernzerhof (PBE) function [26,42,43,44]. The DFT method, which is based on GGA with single-electron approximation, is useful in characterizing a device’s electronic structure. This method is effective in modeling the electronic behavior of a device by doping the carriers with a strongly screened electron–electron interaction [45]. The k-point meshes in the Brillouin zone are set to Monkhorst–Pack 7 × 1 × 145 for the electrode and the central region. The temperature and real-space mesh cutoff are 300 K and 30 Ha, respectively. The boundary conditions for the x, y, and z axes are Neumann, Periodic, and Dirichlet, respectively. The z-axis is always the path of transport.

## 4. Conclusions

In the current work, the performance of the sub-5 FETs was first examined. With the proper UL structure, the I_on_, τ, and PDP of the p-type 3 (5) FETs satisfy the ITRS HP criteria along the arm-(zig-)directions. After considering the NC effect and UL, the p-type 3 FETs can meet ITRS HP criteria along both arm- and zig-directions. Therefore, ML selenene has the potential to be used as a channel material for HP devices and extend Moore’s Law down to the 3 nm scale.

## Figures and Tables

**Table 1:** Benchmark of the ballistic performance of the p-type sub-5 FETs (along the armchair direction) against the ITRS 2013 requirements for HP transistors of the next decades.

	L_g_ (nm)	*L*_UL_ (nm)	SS (mV/dec)	I_off_ (µA/µm)	I_on_ (µA/µm)	I_on_/I_off_	C_t_	τ (ps)	PDP
							(fF/µm)		(fJ/µm)
p-type	5	0	-	0.1	0.38	-	-	-	-
HP		1	123.18	0.1	1416.15	1.42 × 10^4^	0.46	0.21	0.19
		2	100.39	0.1	1717.81	1.72 × 10^4^	0.41	0.15	0.17
	3	0	-	0.1	-	-	-	-	-
		1	-	0.1	-	-	-	-	-
		2	168.15	0.1	153.80	1.54 × 10^3^	0.29	1.22	0.12
		3	117.19	0.1	1010.32	1.01 × 10^4^	0.24	0.15	0.10
	1	0	-	0.1	-	-	-	-	-
		1	-	0.1	-	-	-	-	-
		2	-	0.1	-	-	-	-	-
		3	284.49	0.1	7.86	7.86 × 10^1^	0.13	10.41	0.05
		4	178.68	0.1	157.96	1.58 × 10^3^	0.12	0.47	0.05
ITRS HP 2028	5.1	-	-	0.1	900	9.00 × 10^3^	0.6	0.423	0.24

L_g_: gate length; *L*_UL_: underlap length; SS: subthreshold swing; I_off_: off-state current; I_on_: on-state current; C_t_: total capacitance; τ: delay time; and PDP: power dissipation.

**Table 2:** Benchmark of the ballistic performance of the p-type sub-5 FETs (along the zigzag direction) against the ITRS 2013 requirements for HP transistors of the next decades.

	L_g_ (nm)	*L*_UL_ (nm)	SS (mV/dec)	I_off_ (µA/µm)	I_on_ (µA/µm)	I_on_/I_off_	C_t_	τ (ps)	PDP
							(fF/µm)		(fJ/µm)
p-type	5	0	152.09	0.1	-	-			
HP		1	107.77	0.1	1269.00	1.27 × 10^4^	0.68	0.34	0.28
		2	149.67	0.1	69.20	6.92 × 10^2^	0.92	8.51	0.38
	3	0		0.1	-	-			
		1		0.1	-	-			
		2	299.46	0.1	571.02	5.71 × 10^3^	0.74	0.83	0.30
		3		0.1	-	-			
	1	0	-	0.1	-	-	-	-	-
		1	-	0.1	-	-	-	-	-
		2	-	0.1	-	-	-	-	-
		3	226.47	0.1	17.06	1.71 × 10^2^	0.20	7.67	0.08
		4	-	0.1	-	-			
ITRS HP 2028	5.1	-	-	0.1	900	9.00 × 10^3^	0.6	0.423	0.24

L_g_: gate length; *L*_UL_: underlap length; SS: subthreshold swing; I_off_: off-state current; I_on_: on-state current; C_t_: total capacitance; τ: delay time; and PDP: power dissipation.

## Data Availability

Data openly available in a public repository.

## Supplementary Materials

The following supporting information can be downloaded at: https://www.mdpi.com/article/10.3390/molecules28145390/s1. Figure S1: Transfer characteristics of the *n*- and *p*-type 5 FETs with varying doping concentrations along arm- and zig-directions; Figure S2: LDOS and spectrum current of the *p*-type 5 FETs with *L*_UL_ = 1 nm at the on-, intermediate- and off-state; Figures S3 and S4: the τ and PDP vs. L_g_ in the *p*-type sub-5 FETs with different UL along the arm- and zig-directions; Figure S5: A schematic presentation of the ML selenene FETs with the ferroelectric layers; Transfer characteristics of the *p*-type 5 FETs at *L*_UL_ = 2 nm with and without NC dielectric; Tables S1–S3: Summary of *I*_on_ and *SS* values of the *p*-type sub-5 FETs for the HP application between with and without NC dielectric along the arm- and zig-direction.
