# Supplementary material for: Sub-5 nm Gate-Length Monolayer Selenene Transistors
Source: Molecules. 2023 Jul 13;28(14):5390. doi: 10.3390/molecules28145390 (PMC10385583; doi:10.3390/molecules28145390)
Supplement: Supplementary file 1 [file molecules-28-05390-s001.zip › molecules-2456759-supplementary.pdf]

Supplementary Materials

Sub-5 nm Gate-Length Monolayer Selenene Transistors

Qiang Li [1,*], Xingyi Tan [2], Yongming Yang [1], Xiaoyong Xiong [1], Teng Zhang [1] and Zhulin Weng [1]

[1] College of Intelligent Systems Science and Engineering, Hubei Minzu University, Enshi 445000, China; 2009006@hbmzu.edu.cn (Y.Y.); 2002009@hbmzu.edu.cn (X.X.); hbmyzt@hbmzu.edu.cn (T.Z.); 1994006@hbmzu.edu.cn (Z.W.)

[2] Department of Physics, Chongqing Three Gorges University, Chongqing 404100, China; tanxy@sanxiau.edu.cn

* Correspondence: 2012002@hbmzu.edu.cn

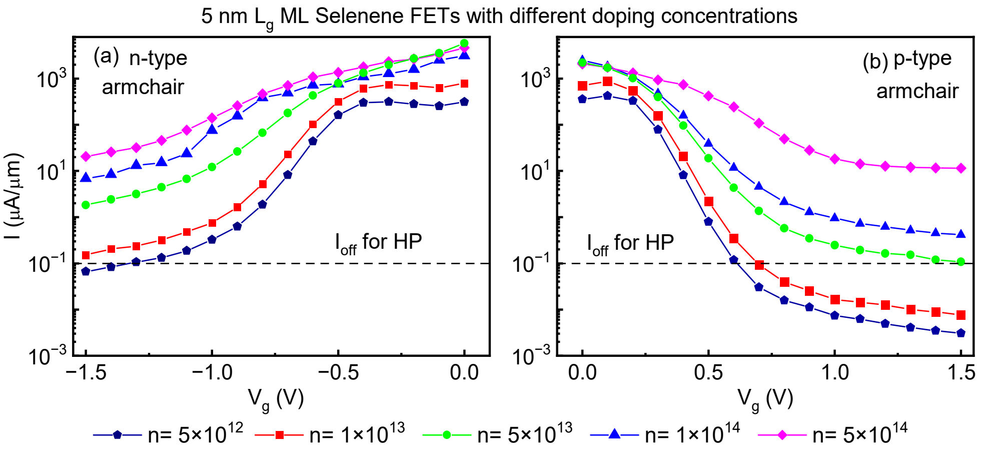

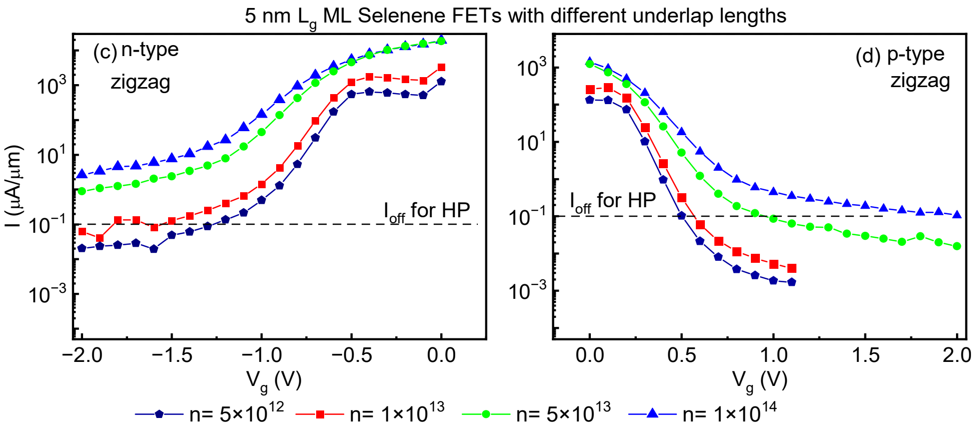

Figure S1. Transfer characteristics of p-type ML selenene FETs with L_g = 5 nm for different sources and drain doping concentrations of electron (N_e) and hole (N_h) (a-b) along the armchair direction and (c-d) zigzag direction.

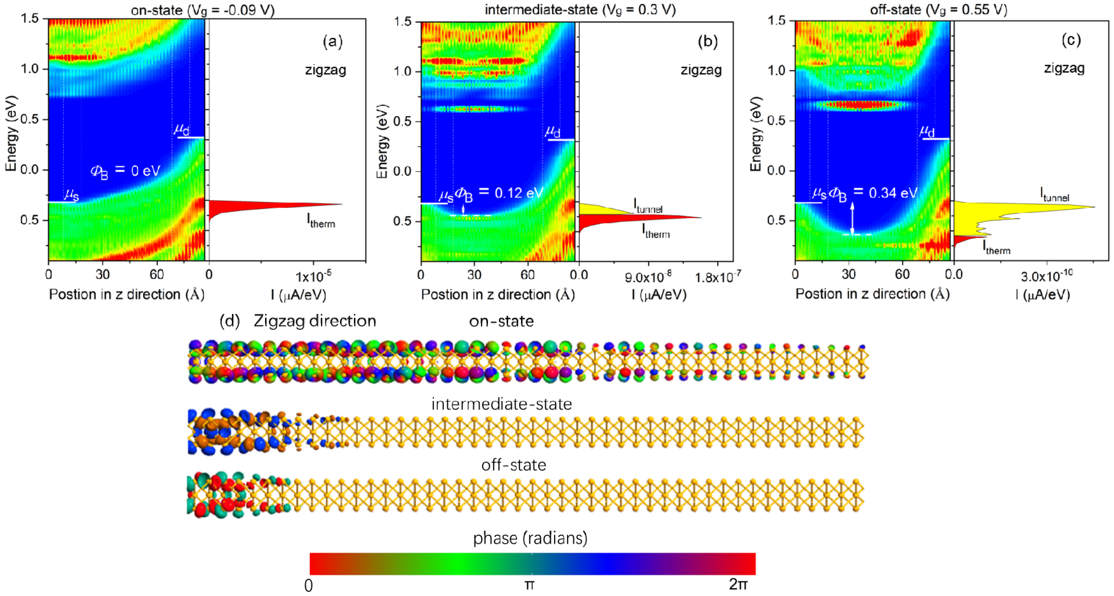

Figure S2. (a-c) LDOS and spectrum current of the 5 nm L_g p-type ML selenene FETs with L_{UL} = 1 nm at the on-, intermediate- and off-state for the HP applications along the zigzag direction. $\mu_{s(d)}$ is the electrochemical potentials of the source(drain), respectively. Φ_B is the activation energy height. (d) Transmission eigenstates of the on-, intermediate-, and off-state for the 5 nm L_g p-type ML selenene FETs with L_{UL} = 1 nm at E = -0.38 eV. The isovalue is 0.05 a.u.

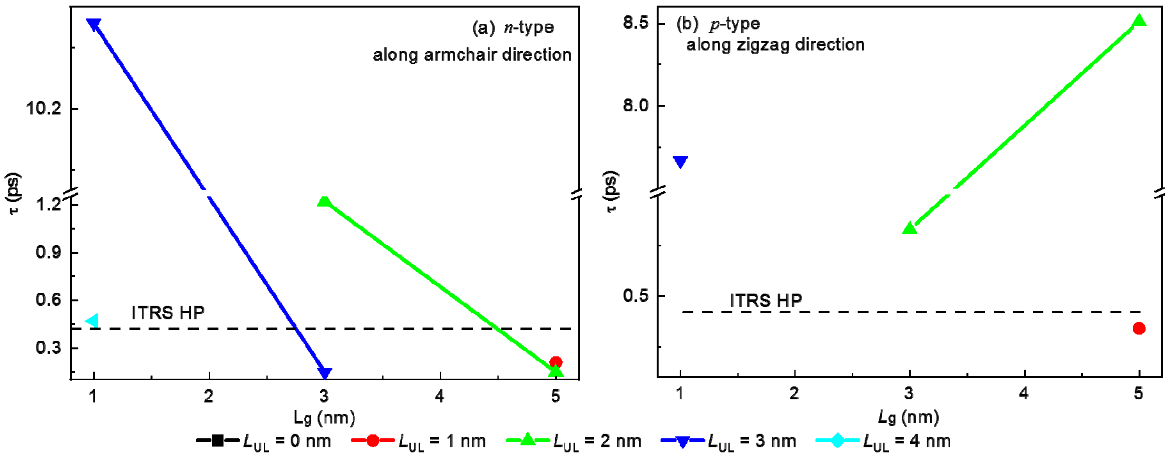

Figure S3. τ versus L_g in the sub-5 FETs with different underlap lengths (a) along the armchair direction (b) along the zigzag direction.

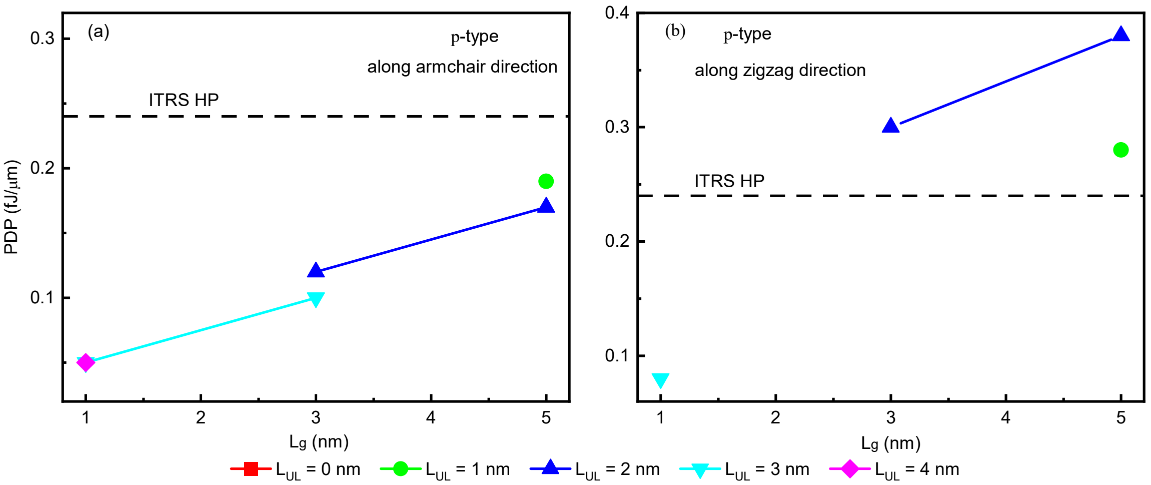

Figure S4. PDP as a function of L_g in the sub-5 FETs with different underlap lengths for the HP applications along the (a) armchair (b) zigzag direction, respectively. Black dashed lines represent the PDP in the 2028 horizon of ITRS 2013 for the HP application.

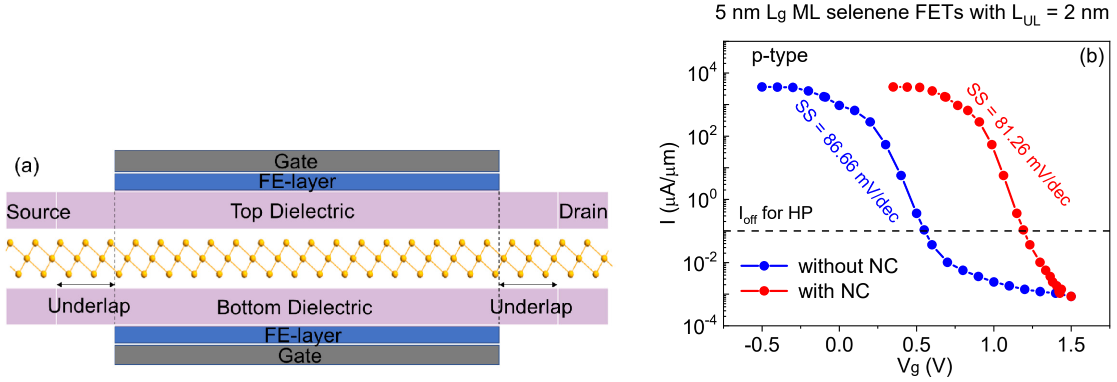

Figure S5. (a) Schematic view of the ML selenene FETs with the ferroelectric layer. (b) Transfer characteristics of the 5 FETs at L_{UL} = 2 nm with and without negative capacity dielectric.

Table S1. Comparison of the ballistic performance of the p-type sub-5 FETs (along the armchair and zigzag direction) against the ITRS 2013 requirements for HP transistors of the next decades.

L_g(nm)	Direction	SS (mV/dec)	I_{on}(μA/μm)	I_{on}/I_{off}	C_t (fF/μm)	τ (ps)	PDP (fJ/μm)
5	Armchair	100.39	1717.81	1.72×10^4	0.41	0.15	0.17
	Zigzag	107.77	1269.00	1.27×10^4	0.68	0.34	0.28
3	Armchair	117.19	1010.32	1.01×10^4	0.24	0.15	0.10
	Zigzag	299.46	571.02	5.71×10^3	0.74	0.83	0.30
1	Armchair	178.68	7.86	7.86×10^1	0.13	10.41	0.05
	Zigzag	226.47	17.06	1.71×10^2	0.20	7.67	0.08
ITRS HP 2028	5.1	-	900	9.00×10^3	0.6	0.423	0.24

L_g: gate length; SS: subthreshold swing; I_{off}: off-state current; I_{on}: on-state current; C_t: total capacitance; τ: delay time; and PDP: power dissipation.

Table S2. Comparison of the I_{on} and SS values of the p-type sub-5 FETs for the HP application between with and without NC dielectric (along the armchair direction).

	L_g (nm)	L_{UL} (nm)	without NC SS (mV/dec)	without NC I_{on} (μA/μm)	with NC SS (mV/dec)	with NC I_{on} (μA/μm)	with NC C_t (fF/μm)	with NC τ (ps)	with NC PDP (fJ/μm)
HP	5	0	-	-	-	-	-	-	-
		1	123.18	1416.15	98.21	3009.96	0.54	0.11	0.22
		2	100.39	1717.81	81.26	3202.95	0.49	0.10	0.20
	3	0	-	-	-	-	-	-	-
		1	-	-	-	-	-	-	-
		2	168.15	153.80	117.27	1421.48	0.45	0.20	0.18
		3	117.19	1010.32	96.84	1735.84	0.29	0.11	0.12
	1	0	-	-	-	-	-	-	-
		1	-	-	-	-	-	-	-
		2	-	-	-	-	-	-	-
		3	284.49	7.86	197.07	202.57	0.18	0.57	0.07
		4	178.68	157.96	125.81	616.88	0.19	0.20	0.08
ITRS	5.1			900		900	0.6	0.423	0.24

L_g: gate length; L_{UL}: underlap length; SS: subthreshold swing; I_{off}: off-state current; I_{on}: on-state current; C_t: total capacitance; τ: delay time; and PDP: power dissipation.

Table S3. Comparison of the I_{on} and SS values of the p-type sub-5 FETs for the HP application between with and without NC dielectric (along the zigzag direction).

	L_g (nm)	L_{UL} (nm)	without NC SS (mV/dec)	without NC I_{on} (μA/μm)	with NC SS (mV/dec)	with NC I_{on} (μA/μm)	with NC C_t (fF/μm)	with NC τ (ps)	with NC PDP (fJ/μm)
HP	5	0	152.09	-	-	-	-	-	-
		1	107.77	1269.00	91.09	1914.77	0.74	0.25	0.30

		2	149.67	69.20	106.92	1264.07	1.34	0.68	0.55
	3	0		-	-	-	-	-	-
		1		-	-	-	-	-	-
		2	299.46	571.02	129.59	1070.50	0.51	0.31	0.21
		3	-	-	-	-	-	-	-
	1	0	-	-	-	-	-	-	-
		1	-	-	-	-	-	-	-
		2	-	-	-	-	-	-	-
		3	226.47	17.06	168.38	398.70	0.37	0.59	0.15
		4	-	-	-	-	-	-	-
ITRS	5.1			900		900	0.6	0.423	0.24

L_g: gate length; L_{UL}: underlap length; SS: subthreshold swing; I_{off}: off-state current; I_{on}: on-state current; C_t: total capacitance; τ: delay time; and PDP: power dissipation.
